# USF1 deficiency alleviates inflammation, enhances cholesterol efflux and prevents cholesterol accumulation in macrophages

**DOI:** 10.1186/s12944-018-0930-2

**Published:** 2018-12-13

**Authors:** Maija Ruuth, Jarkko Soronen, Essi Kaiharju, Krista Merikanto, Julia Perttilä, Jari Metso, Miriam Lee-Rueckert, Marja-Riitta Taskinen, Petri T. Kovanen, Katariina Öörni, Vesa M. Olkkonen, Matti S. Jauhiainen, Pirkka-Pekka Laurila

**Affiliations:** 1 Wihuri Research Institute, FI-00290 Helsinki, Finland; 2 Research Program Unit, University of Helsinki, FI-00014 Helsinki, Finland; 3 Genomics and Biomarkers Unit, National Institute for Health and Welfare, FI-00251 Helsinki, Finland; 4 Minerva Foundation Institute for Medical Research, Tukholmankatu 8, 00290 Helsinki, Finland; 5 Diabetes and Obesity Research Program, University of Helsinki, FI-00014 Helsinki, Finland; 6 Department of Anatomy, Faculty of Medicine, University of Helsinki, FI-00014 Helsinki, Finland; 7 Department of Medical and Clinical Genetics, University of Helsinki, FI-00014 Helsinki, Finland; 8 Institute for Molecular Medicine Finland, FIMM, FI-00251 Helsinki, Finland

**Keywords:** *USF1*, High density lipoproteins, Cholesterol efflux, Cholesterol accumulation, Macrophage, Hepatocyte, Inflammation

## Abstract

**Background:**

The focus of studies on high-density lipoproteins (HDL) has shifted from HDL-cholesterol (HDL-C) to HDL function. We recently demonstrated that low *USF1* expression in mice and humans associates with high plasma HDL-C and low triglyceride levels, as well as protection against obesity, insulin resistance, and atherosclerosis. Here, we studied the impact of USF1 deficiency on HDL functional capacity and macrophage atherogenic functions, including inflammation, cholesterol efflux, and cholesterol accumulation.

**Methods:**

We used a congenic *Usf1* deficient mice in C57Bl/6JRccHsd background and blood samples were collected to isolate HDL for structural and functional studies. Lentiviral preparations containing the *USF1* silencing shRNA expression vector were used to silence *USF1* in human THP-1 and Huh-7 cells. Cholesterol efflux from acetyl-LDL loaded THP-1 macrophages was measured using HDL and plasma as acceptors. Gene expression analysis from *USF1* silenced peritoneal macrophages was carried out using Affymetrix protocols.

**Results:**

We show that *Usf1* deficiency not only increases HDL-C levels in vivo, consistent with elevated ABCA1 protein expression in hepatic cell lines, but also improves the functional capacity of HDL particles. HDL particles derived from *Usf1* deficient mice remove cholesterol more efficiently from macrophages, attributed to their higher contents of phospholipids. Furthermore, silencing of *USF1* in macrophages enhanced the cholesterol efflux capacity of these cells. These findings are consistent with reduced inflammatory burden of USF1 deficient macrophages, manifested by reduced secretion of pro-inflammatory cytokines MCP-1 and IL-1β and protection against inflammation-induced macrophage cholesterol accumulation in a cell-autonomous manner.

**Conclusions:**

Our findings identify USF1 as a novel factor regulating HDL functionality, showing that USF1 inactivation boosts cholesterol efflux, reduces macrophage inflammation and attenuates macrophage cholesterol accumulation, linking improved macrophage cholesterol metabolism and inflammatory pathways to the antiatherogenic function of USF1 deficiency.

## Introduction

Low level of high-density lipoprotein cholesterol (HDL-C) is a major independent risk factor for atherosclerotic cardiovascular disease [1]. However, recent studies have shown that raising HDL-cholesterol (HDL-C) levels by pharmacological agents does not translate into better clinical outcomes. The HDL-C raising agent niacin and CETP inhibitors, when administered on top of statins, both failed to reduce cardiovascular events despite markedly increasing HDL-C levels [2–4]. Furthermore, Mendelian randomization studies using complex genetics have shown that variants associated exclusively with high HDL-cholesterol levels, are not associated with reduction in cardiovascular outcomes [5]. These findings suggest that HDL-C is unlikely to be causally related to cardiovascular disease. Thus, research efforts have shifted from HDL-C levels to HDL function.

The canonical functions of HDL particles include their anti-inflammatory and antioxidative properties, as well as their role in the initiation of reverse cholesterol transport [6]. Cholesterol efflux from cells to circulation is the first and rate-limiting step in reverse cholesterol transport. Recently, strategies to measure cholesterol efflux capacity of apolipoprotein B-depleted plasma have been successfully used in clinical studies, revealing inverse correlations between cholesterol efflux capacity and prevalent coronary artery disease, even stronger than HDL-C levels [7, 8]. Indeed, the levels of circulating HDL-C do not strictly reflect the cholesterol molecules released from the macrophage foam cells typical of atherosclerotic lesions, neither do they exert any of the anti-atherogenic activities of HDL [9]. Thus, further understanding of the factors affecting cholesterol efflux might reveal a higher atheroprotective potential than HDL-C targeting agents.

The upstream stimulatory factor 1 (USF1) was originally associated with familial combined hyperlipidemia [10] with subsequent associations with lipid levels [11] [12, 13], coronary atherosclerosis [14], and acute cardiovascular events [15]. We recently showed that inactivation of USF1 in mice protects against atherosclerosis, insulin resistance, obesity, and hepatic steatosis [16]. The beneficial metabolic phenotype of this mouse model was linked to increased whole-body energy expenditure and brown adipose tissue activity. Furthermore, *Usf1* deficiency corrected diet-induced dyslipidemia in mice, manifested by reduced triglycerides and elevated levels of HDL-C. The atheroprotective role of *USF1* deficiency was also observed in humans, in whom individuals having a *USF1* mRNA expression reducing allele displayed elevated HDL-C and reduced triglyceride levels, as well as reduced atherosclerosis in coronary arteries, also featuring fewer calcified plaques [16]. However, whether the elevated HDL-C was associated with an improved functionality of HDL particles was not addressed in the previous study. We now investigate whether USF1 affects pathways involved in macrophage cholesterol accumulation, with particular focus on cholesterol efflux, the rate-limiting step in reverse cholesterol transport, and whether the elevated HDL-C of *Usf1* deficient animals translates into improved HDL function.

## Materials and methods

### In vivo experiments

#### Animals

We use a congenic strain in C57Bl/6JRccHsd background in our studies with littermate controls. All animal procedures were reviewed and approved by the local animal care committee and local government authorities. The generation of *Usf1* deficient mice has been previously described [16].

#### Isolation and composition of mouse HDL

HDL was isolated from pooled mice serum samples by sequential ultracentrifugation using Table-Top ultracentrifuge (Beckmann Optima TL, USA) and KBr for density adjustment. Serum samples were first adjusted to the density (d) of 1.019 g/mL and the centrifuge tube filled with a d = 1.019 g/mL KBr solution to the total volume of 3 mL. The samples were centrifuged at + 5 °C for 2 h at relative centrifugal field 500,000 x G. After centrifugation very low and intermediate density lipoproteins (VLDL and IDL) were recovered in the top 1 mL fraction and the bottom was adjusted to the density of 1.063 g/mL using solid KBr, filled to 3 mL with d = 1.063 g/mL KBr solution and centrifuged (+ 5 °C, 3 h, 500,000 x G). The top 1 mL fraction contained low density lipoproteins (LDL). To get the total mouse HDL fraction the LDL infranatant fraction was adjusted with solid KBr to the density of 1.21 g/mL, the vials filled with KBr 1.21 g/mL density solution and then centrifuged (+ 5 °C, 18 h, 500,000 x G). Total HDL was obtained in top 1 mL fraction. The isolated HDL was dialyzed against phosphate-buffered saline (PBS, pH 7.4) and stored at + 4 °C before analysis. Isolated HDL was analyzed for lipids and APOA1 using the methods described below.

#### Analysis of lipid and apoA-I concentration

Isolated HDL particles were analyzed for total cholesterol (CHOD-PAP 1489232 kit; Roche Diagnostics GmbH), choline-containing phospholipids (990–54,009; Wako Chemicals GmbH) and triglycerides (GPO-PAP 1488872 kit; Roche Diagnostics GmbH) using fully enzymatic methods. Mouse apolipoprotein A-I (apoA-I) was quantified by a sandwich enzyme-linked immunosorbent assay (ELISA) as described [17].

#### Gene expression analysis from *USF1* silenced peritoneal macrophages

RNA was extracted using the RNeasy Mini Kit (Qiagen) according to manufacturer’s instructions. Quality of RNA was analyzed using the Bioanalyzer platform (Agilent Technologies). Two micrograms of total RNA were treated according to standard Affymetrix eukaryotic RNA labeling protocols (Affymetrix, Santa Clara, CA). Fifteen micrograms of biotin labeled cRNA was fragmented according to Affymetrix eukaryotic sample protocol. Hybridization, staining, and washing of the Affymetrix HG-U133_Plus_2 Arrays were performed using the Affymetrix Fluidics Station 450 and Hybridization Oven 640 under standard conditions. The raw data were processed using the GCRMA-normalization method [18].

#### Cholesterol accumulation in mouse peritoneal macrophages

Mouse peritoneal macrophages were collected from 8 weeks old female mice. Mice were pretreated 4 days with 500 μL thioglycollate (BD, BLL™ Thioglycollate Medium Brewer Modified). Macrophages were collected in 1% BSA in PBS and cultured in D-MEM (Sigma) supplemented with 20% FBS and 1% penicillin/streptomycin. Cells were cultured for 4 days before experimentation to remove the thioglycollate induced inflammation. For cholesterol loading peritoneal macrophages were incubated with acetylated LDL for 16 h. Some samples were pre-treated with 10 ng/mL of LPS *(Salmonella Minnesota R595)* for 24 h before loading. Lipids were extracted from the cells with organic solvent using n-Hexane and isopropanol, 3:2 (*v*/v). After evaporation of the organic solvent under nitrogen the residual lipids were dissolved in methanol. Total cholesterol was measured with commercial kit (CHOD-PAP # 1489232, Roche Diagnostics GmbH, Mannheim, Germany). Remaining cells after lipid isolation were collected and total protein was measured for adjustment of the final results.

### In vitro experiments

#### Silencing of USF1 in vitro

The most effective *USF1* silencing shRNA was screened in immortalized human hepatocytes (IHH). IHH were grown in William’s E medium (GIBCO-Life Technologies, Carlsbad, CA) containing 10% fetal bovine serum (FBS), 2 mM L-glutamine, 100 U/mL penicillin, 100 μg/mL streptomycin, 100 nM (20 mU/mL) insulin, and 50 nM dexamethasone (Sigma-Aldrich, St. Louis, MO) on CellBIND® Surface cell culture plates (Corning, Corning, NY). The human monocytic cell line, THP-1 and human hepatocellular carcinoma cell line, HuH-7 were cultured in RPMI (THP-1) or DMEM (HuH-7) medium supplemented with 10% fetal bovine serum and penicillin/streptomycin and to THP-1 media 25 mM HEPES was added. For transduction, 20,000 THP-1 cells seeded on 24-well plates or ~ 80% confluent HuH-7 cells in 12-well plates were treated for 24 h with SIGMA MISSION lentiviral preparations containing either the control shRNA expression vector (MISSION® pLKO.1-puro Non-Target shRNA) or the *USF1* silencing shRNA expression vector (233475) at a MOI of ~ 1. Cells were selected with 6 μg/mL of puromycin for 14 days and were then used for cholesterol efflux assays.

Cell lines were found to be free of *Mycoplasma* contamination.

#### Cholesterol efflux from THP-1 macrophages

THP-1 human monocytes (ATCC, Manassas, VA) were grown at 37 °C in suspension culture in RPMI 1640 medium supplemented with 10% FBS, 25 mM HEPES, and 1% penicillin/streptomycin. Differentiation into macrophages was performed using 100 nM phorbol myristate acetate (PMA, Sigma) for 3 days. After this the differentiated macrophages were washed twice with PBS and loaded by incubation in the presence of [^3^H]cholesteryl oleate-labeled acetyl-LDL (25 μg of protein/well) in RPMI 1640 supplemented with 5% (*v*/v) lipoprotein-deficient serum, 10 mM HEPES, pH 7.4, and penicillin/streptomycin for 48 h and without PMA. The cholesterol-loaded macrophage cells were then washed with PBS, and 2% serum or HDL (as determined by 25 μg/mL HDL protein) derived from either *Usf1*^***−/−***^ or *Usf1*^*+/+*^ mice were added as cholesterol acceptors. After 24 h incubation, the growth medium was collected, and radioactivity was determined by liquid scintillation counting. The cell layer was washed with PBS, followed by addition of 0.2 M NaOH to lyse the cells and further incubated for 24 h at + 4 °C, after which the radioactivity was assessed. Wells incubated without added acceptors were used as controls. Cholesterol efflux is expressed as dpm in medium divided by the cell protein content (unit used for the efflux: dpm/μg cell protein).

#### Western blot analysis of ABCA1

Cells were lysed on ice with buffer (10 mM HEPES, 150 mM NaCl, 0,5 mM MgCl_2_, 10% Glycerol, 0,5% Triton X-100, 0,5% deoxycholate, pH 7.4) containing protease inhibitors. Protein concentrations were determined, and equal amount of proteins were loaded on 4–12% SDS-PAGE gel (Bio-RAD) in sample buffer containing β-mercaptoethanol without heating. After electrophoresis the separated proteins in gel were electro-transferred to nitrocellulose membrane. Membrane was blocked with 5% milk TBS-T for 1 h and after washing immunostaining was performed with primary antibody (ABCA1, Bio-RAD and β-actin, Abcam), washed and followed by incubation with Chemiluminence (Daco) and IRDye 680RD (LI-COR) secondary antibodies. Immunoreactive proteins were detected with Super Signal West Femto Maxium Sensitivity Substrate (ThermoFisher Scientific) using chemiluminesence and infrared imaging system (LI-COR). Result were calculated with Image Studio Lite program and the ABCA1 signal was adjusted to β*-*actin.

#### Cholesterol esterification measurements

*USF-1*-silenced and control THP-1 macrophages were incubated with or without acetylated LDL (acLDL, 50 μg/ml) in serum-free media for 24 h. Cells were washed 3 times with PBS and incubated with [^3^H]-oleic acid-BSA complexes in the serum-free medium for 2 h, washed 3 times with PBS, and lipids were extracted with hexane/isopropanol (3:2, *v*/v). Samples of the cell suspensions were taken for protein determination and from the extracted lipids [^3^H]-oleate incorporation into cholesteryl esters was analyzed using thin layer chromatography (TLC). The extracted lipids were separated by TLC in n-heptane/isopropyl ether/glacial acetic acid/methanol (60:40:4:2, vol/vol). Spots corresponding to cholesteryl esters (CE) were scraped, the radioactivity measured by scintillation counting and the CE counts per total cell protein calculated.

For measuring the distribution of [^14^C]-cholesterol between unesterified cholesterol (C) and CE *USF-1*-silenced and control THP-1 macrophages were incubated with [^14^C]-cholesterol (1 μCi/ml) in serum-free media for 24 h, washed with PBS and chased in serum-free medium for 18 h. Lipids were extracted and separated by TLC in n-hexane/diethyl ether/glacial acetic acid/water (65:15:1:0.25, vol/vol). Spots corresponding to C and CE were scraped, the radioactivity measured by scintillation counting, and the [^14^C]-CE/total [^14^C]-cholesterol and [^14^C]-CE/total cell protein ratios were calculated.

## Results

**Fig. 1:**
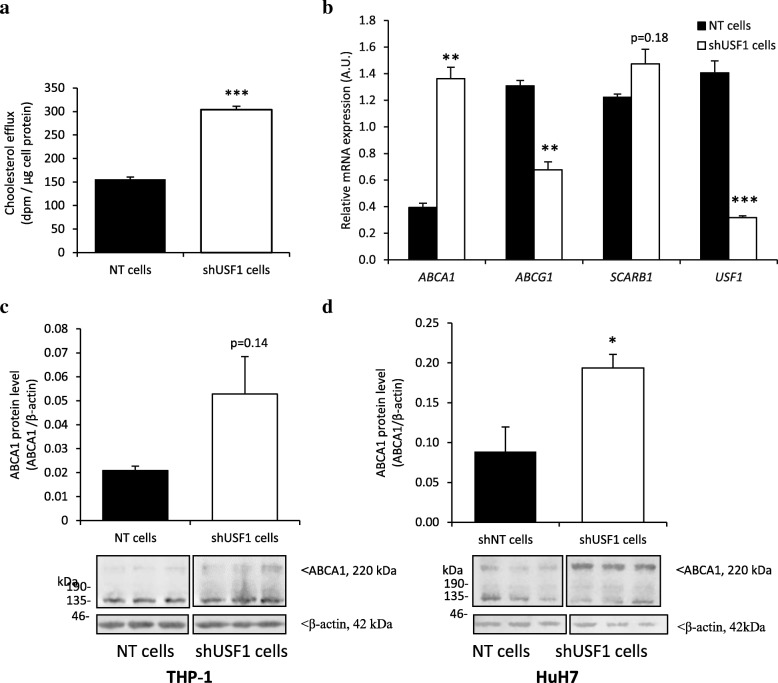
Inactivation of USF1 enhances cholesterol efflux. **a**) Cholesterol efflux from THP-1 macrophages treated with non-targeting (NT) and USF1 silencing lentivirus (shUSF1) to wild-type mouse serum, n=6 per condition from two independent experiments. (**b**) Gene expression of cholesterol transporters relevant for cholesterol efflux in THP-1 macrophages. n=3-4. (**c**) ABCA1 protein expression in THP-1 cells. n=6/6 from two independent experiments. (**d**) ABCA1 protein expression in HuH7 hepatoma cells. n=6/6 from two independent experiments. Molecular weights of 220Kda for ABCA1 and 42 kDa for β-actin are depicted in Panels **c** and **d**. *** *P* < 0.001, ** *P* < 0.01, * *P* < 0.05, for comparisons between groups

As previously shown, the *Usf1*^*−/−*^ mice displayed elevated levels of plasma total cholesterol and phospholipids [16]. Using fast protein liquid chromatography, we established that cholesterol and phospholipid concentrations were elevated in the HDL fractions of *Usf1*^*−/−*^ mice as compared *Usf1*^*+/+*^ mice [16]. In the present study, we first investigated whether USF1 inactivation in human macrophages affects cholesterol efflux from macrophage foam cells. We silenced *USF1* in THP-1 macrophages using shRNA, achieving 79% silencing, and observed significant increase in cholesterol efflux capacity from these cells (Fig. 1a). This could be mediated by ABCA1, whose mRNA expression was upregulated by about 3.2-fold in the THP-1 cells subjected to USF1 knock-down (Fig. 1b) whereas the expression of *ABCG1* was downregulated (52%), and that of SCARB1 (commonly known as SR-BI) remained unaltered. In Western blot analyses, consistent with higher *ABCA1* transcript abundance, ABCA1 displayed a trend of increase in *USF1* silenced THP-1 macrophages (Fig. 1c). Interestingly, we also detected a significant increase of ABCA1 protein in human hepatoma cells subjected to *USF1* knock-down (HuH7; Fig. 1d). Since USF1 has previously been identified as a repressor of ABCA1 [19], our results suggest that USF1 inactivation could boost both cholesterol efflux capacity of macrophages as well as enhance nascent HDL particle secretion from the liver, contributing to the HDL elevation observed in *Usf1*^*−/−*^ mice [16].

**Fig. 2:**
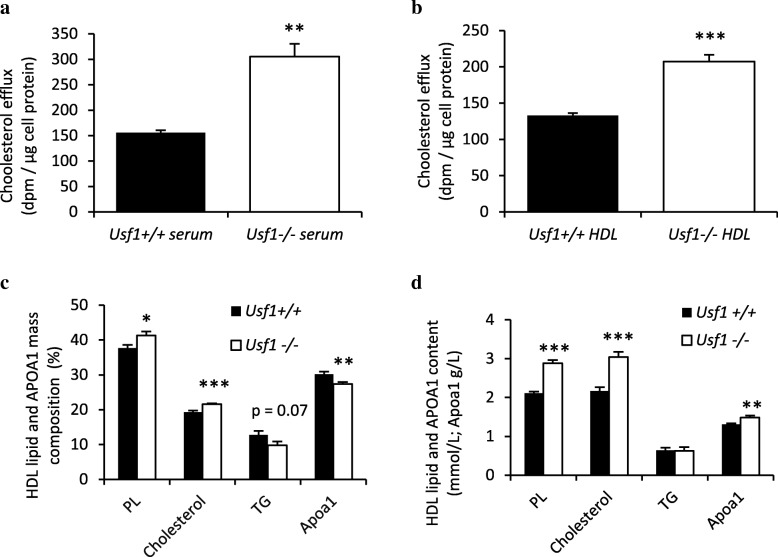
USF1 deficiency improves acceptor capacity of serum and HDL particles, enriched with phospholipids (PL). (**a**) Cholesterol efflux from THP-1 macrophages to serum derived from Usf1+/+ and Usf1-/- mice. n=5 for each column. Serum samples from five individual Usf1+/+ and Usf1-/- mice were analyzed in two independent experiments. (**b**) Cholesterol efflux from THP-1 macrophages to HDL particles derived from Usf1+/+ (n=7 mice, pooled HDL) and Usf1-/- mice (n=8 mice, pooled HDL), n=6 parallel wells for THP-1 cells in two independent experiments. (**c**) Mass composition (%) of HDL particles isolated from Usf1+/+ and Usf1-/- mice. n=11/9. (**d**) HDL particle content (mmol/L for lipid and g/L for APOA1), n=11/9. *** *P* < 0.001, ** *P* < 0.01, * *P* < 0.05, for comparisons between groups

In addition to studying the impact of USF1 on the key cholesterol transporters, we also studied its effects on cholesterol acceptors. Serum derived from *Usf1*^*−/−*^ mice displayed enhanced capacity to induce cholesterol efflux from THP-1 foam cells as compared to *Usf1*^*+/+*^ serum (Fig. 2a). Interestingly, when the experiment was repeated with HDL isolated from mouse sera, the acceptor capacity of *Usf1*^*−/−*^ derived HDL was superior to that of *Usf1*^*+/+*^ HDL (Fig. 2b), explaining the enhanced serum facilitated removal of cholesterol from macrophage foam cells. Thus, both enhanced cholesterol transporter expression and cholesterol acceptor functionality appear to boost the first step of macrophage-specific reverse cholesterol transport in conditions of USF1 deficiency.

Elevated levels of HDL-C are associated with improved HDL lipid composition in humans [20, 21], and increased HDL phospholipid content has been reported to confer better cholesterol acceptor properties to HDL particles [22, 23]. We next studied whether HDL composition accounts for the higher cholesterol efflux efficiency resulting from USF1 inactivation. The HDL particles derived from *Usf1*^*−/−*^ mice were enriched in phospholipids and cholesterol while the proportional amount of apolipoprotein A1 (APOA1) was decreased (Fig. 2c), indicating a higher phospholipid/APOA1 mass ratio in HDL. The higher proportion of phospholipids (PL) provides a plausible explanation to the elevated cholesterol acceptor capacity of *Usf1*^*−/−*^ derived HDL particles. Importantly, the concentrations of phospholipids, cholesterol, and APOA1 were elevated in the HDL fraction derived from *Usf1*^*−/−*^ mice (Fig. 2d). Together, these findings explain the functional superiority of *Usf1*^*−/−*^ HDL and serum as acceptors in cholesterol efflux.

**Fig. 3:**
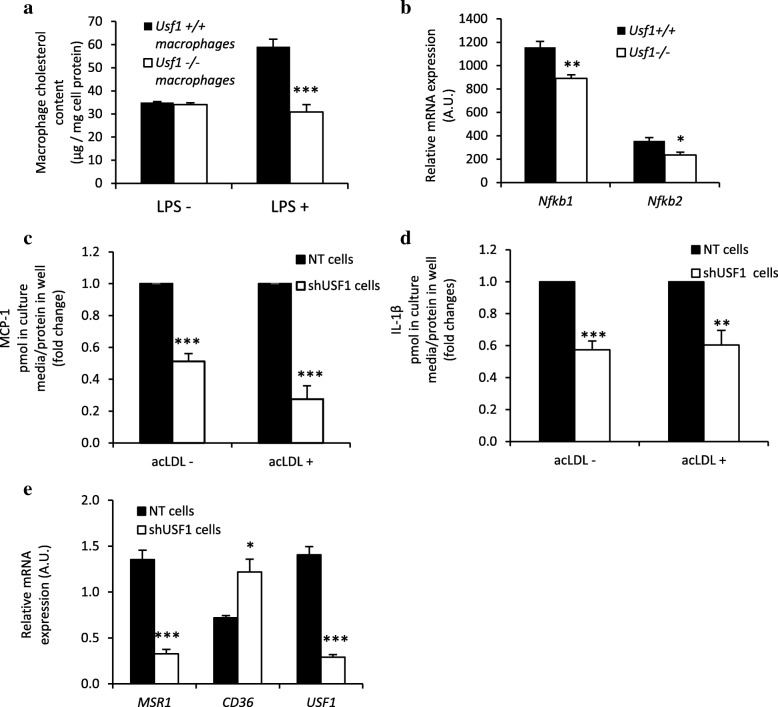
USF1 deficiency attenuates LPS-induced proinflammatory responses of macrophages and protects against inflammation-induced cholesterol accumulation. (**a**) Cholesterol content of mouse peritoneal macrophages incubated without or with LPS. n=4/5/9/8 parallel wells in two independent experiments. (**b**) Expression of Nfkb1 and Nfkb2 (NF-κB) in peritoneal macrophages in the absence of LPS. n=4/4. (**c**) Secretion of MCP-1 from THP-1 macrophages +/- acLDL, n=6 from 2 independent experiments. (**d**) Secretion of IL-1β from THP-1 macrophages +/- acLDL, n=6 from 2 independent experiments. (**e**) Gene expression of cholesterol transporters relevant for cholesterol uptake in THP-1 macrophages in the absence of LPS. n=4 in each column. *** *P* < 0.001, ** *P* < 0.01, * *P* < 0.05, for comparisons between groups

Because increased uptake of modified LDL by macrophages promotes the generation of cholesterol-loaded foam cells [24], an early sign of atherosclerosis, we next examined whether expression of USF1 has an effect on cholesterol accumulation in macrophages. For this aim, we measured the content of cholesterol in peritoneal macrophages from *Usf1*^*+/+*^ and *Usf1*^*−/−*^ mice after incubation of the cells with acetylated LDL in the absence or presence of lipopolysaccharide (LPS), a potent proinflammatory activator of macrophages. We found that while in the absence of LPS there was no difference, in the presence of LPS the cholesterol accumulation induced by acLDL was 48% lower in *Usf1* deficient macrophages as compared to wild type cells (Fig. 3a). *Usf1* deficiency thus confers resistance to LPS-induced proinflammatory response in macrophages. The reduced proinflammatory response in *Usf1* deficient macrophages was also reflected by lower mRNA expression of NF-κB, including both *Nfkb1* and *Nfkb2* (Fig. 3b). Furthermore, secretion of pro-inflammatory cytokines MCP-1 (Fig. 3c) and IL-1β (Fig. 3d) was significantly reduced in *USF1*-silenced THP-1 macrophages when compared to control cells, demonstrating the presence of a cell-autonomous component of the anti-inflammatory effect of USF1 deficiency.

Modified LDL is taken up by several types of scavenger receptors, of which SR-A1 (gene name *MSR1*) and CD36 are responsible for > 75% of its internalization [25]. SR-A1 *(MSR1)* mRNA expression was downregulated by 4.2-fold in USF1 silenced THP-1 macrophages (Fig. 3e), likely explaining the relative resistance conferred by USF1 deficiency to cholesterol accumulation in macrophages observed upon the inflammation-mimicking conditions (LPS exposure). In contrast, the mRNA expression of CD36 was upregulated by 1.7-fold (Fig. 3e). Taken together, by both reducing cholesterol accumulation and enhancing cholesterol efflux, *USF1* deficiency prevents cholesterol accumulation in macrophages, thus attenuating foam cell formation.

**Fig. 4:**
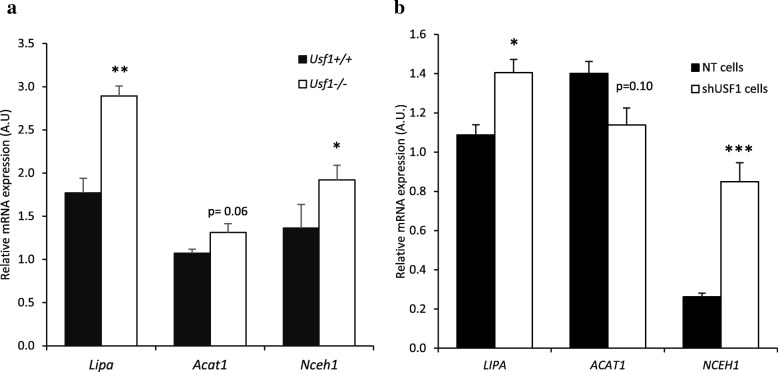
Intracellular cholesterol pathways are upregulated upon USF1 deficiency. (**a**) Lipa, Acat1 and Nceh1 mRNA expression in mouse peritoneal macrophages. n=5/5 (individual animals). (**b**) LIPA, ACAT1 and NCEH1 mRNA expression in THP-1 macrophages. n=4/3; for NCEH n=3 per group. ** *P* < 0.01, * *P* < 0.05, for comparisons between groups

As macrophage cholesterol efflux is determined not only by the quantity and quality of cholesterol acceptors and transmembrane transporters, but also by the function of intracellular lipid-regulatory enzymes, we next investigated the effect of USF1 on intracellular enzymes affecting cholesterol efflux. Lysosomal acid lipase (LAL; gene name *Lipa*) is an enzyme responsible for hydrolysis of cholesterol esters. LIPA has been reported to contribute to cholesterol efflux [26], and overexpression of *Lipa* has been shown to prevent atherogenesis [27, 28]. ACAT1 re-esterifies free cholesterol generated by LIPA to produce cytoplasmic cholesterol ester droplets, and its deficiency results in decreased cholesterol efflux from macrophages [29]. Neutral cholesterol ester hydrolase 1 *(Nceh1)*, located on the cholesterol ester droplets, mediates their hydrolysis in both murine and human macrophages [30], and ablation of *Nceh1* accelerates atherosclerosis [31]. To study these intracellular pathways regulating cholesterol efflux, we conducted genome-wide expression array analysis of peritoneal macrophages isolated from *Usf1*^*+/+*^ and *Usf1*^*−/−*^ mice. This analysis demonstrated that in the USF1 deficient macrophages the expression of *Lipa* was upregulated by 63%, *Acat1* by 23%, and *Nceh1* by 41% (Fig. 4a). In addition, in THP-1 macrophages similar increases in expression upon USF1 silencing were observed for both *NCEH1* and *LIPA*, but not for *ACAT1* (Fig. 4b). In functional analysis, however, measurement of macrophage cholesterol esterification by a radiometric assay showed no significant difference between *USF1* silenced (14,805 ± 1266 dpm/mg protein) and control THP-1 macrophage cells (12,500 ± 2327 dpm/mg protein). We further analyzed the impact of USF1 silencing on the distribution of [^14^]-cholesterol between unesterified and esterified intracellular pools in the THP-1 macrophages in the absence and presence of acLDL loading. These experiments revealed no significant difference in the [^14^C]CE/total C ratio (no acLDL loading: shNT, 0.0549 ± 0.0135 cpm/cpm; shUSF1, 0.0676 ± 0.0316 cpm/cpm, and acLDL loading: shNT, 0.3641 ± 0.0464 cpm/cpm; shUSF1, 0.4374 ± 0.0108 cpm/cpm) or in the [^14^C]CE/total protein ratio (no acLDL loading: shNT, 3.508 ± 0.424 cpm/μg protein; shUSF1, 4.047 ± 0.225 cpm/μg protein, and acLDL loading; shNT, 22.801 ± 7.906 cpm/μg protein; shUSF1, 27.215 ± 3.508 cpm/μg protein). Although we did not observe differences in the ACAT-mediated cholesterol esterification or the distribution of [^14^C[−cholesterol, the enhanced cholesterol efflux and the elevated expression of *ABCA1* mRNA and protein, as well as the altered mRNAs expression of *LIPA* and *NCEH1* imply that the effects of *Usf1* deficiency extend to inducing modulation of intracellular cholesterol pathways which also contribute to the enhancement of macrophage cholesterol efflux.

## Discussion

**Fig. 5:**
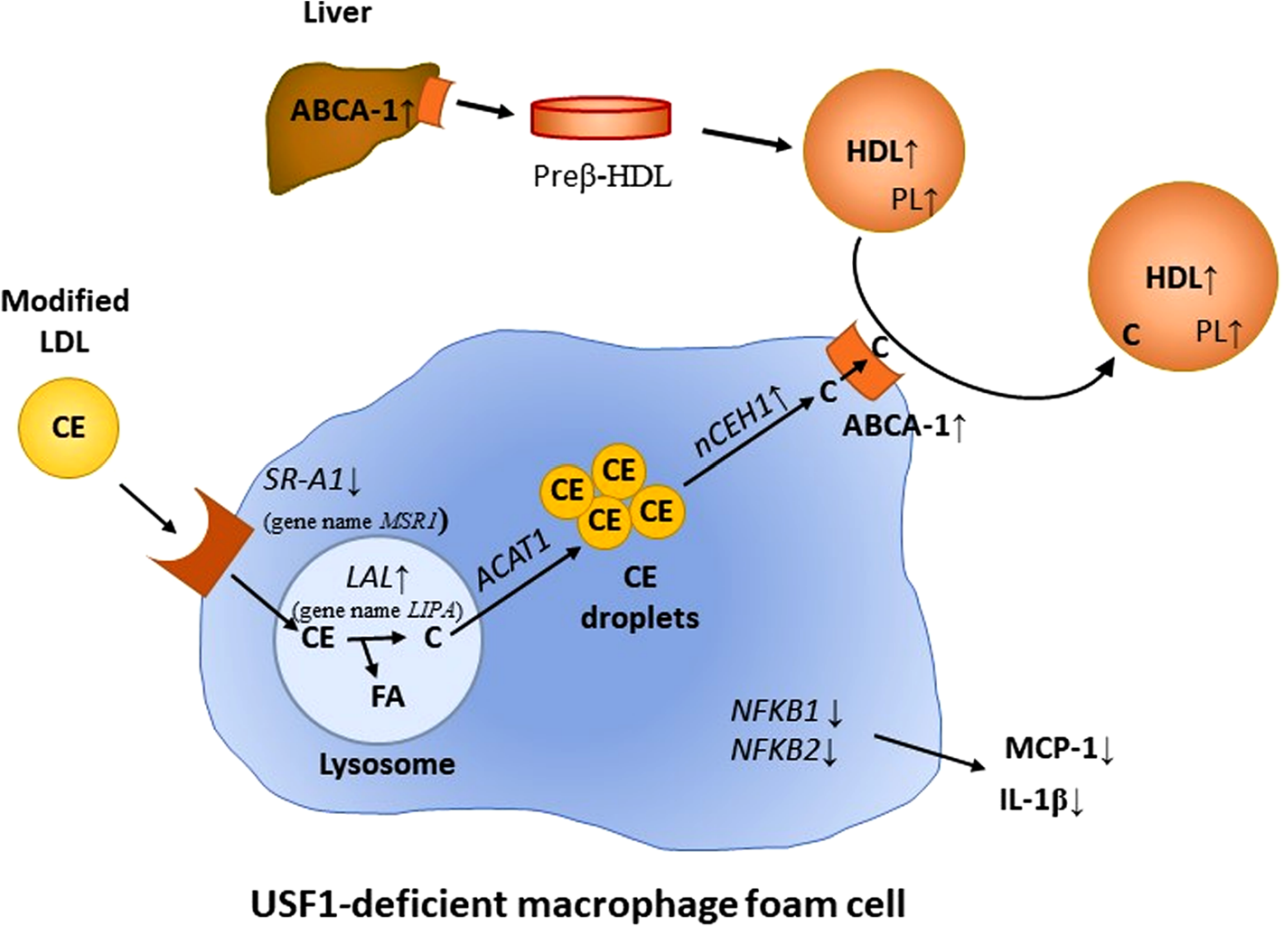
Schematic presentation of the proposed mechanism for the effects of USF1 deficiency on cholesterol flux through a macrophage foam cell. Uptake of cholesteryl esters (CE) present in the core of modified LDL particles promotes the generation of macrophage foam cells. Modified LDL is taken up by scavenger receptors, such as SR-A1. Lack of USF1 resulted in reduced mRNA levels of SR-A1 and attenuated LPS-induced macrophage cholesterol deposition. Intracellular cholesterol flux-regulating enzymes, lysosomal acid lipase (LAL, gene name LIPA), acetyl-CoA cholesterol acyltransferase 1 (ACAT1) and neutral cholesterol ester hydrolase 1 (nCEH1) are key factors modulating macrophage cholesterol metabolism. Deficiency of Usf1 caused an upregulation in expression of NCEH1 and LIPA in both mouse peritoneal macrophages and human THP-1 cells, thereby enhancing intracellular cholesterol flux from lysosomes via CE droplets to plasma membrane. The protein expression of ABCA1, an important cholesterol transporter on macrophage plasma membrane, was also increased due to lack of USF1, further accelerating the removal of cholesterol to cholesterol acceptors (HDL), whose plasma levels were elevated in Usf1 deficient mice (16). The HDL particles derived from Usf1-/- mice had a higher proportion of phospholipids (PL) in HDL further explaining the elevated cholesterol acceptor capacity of Usf1-/- derived HDL particles. ABCA1 protein levels were upregulated in USF1 deficient hepatocytes, which may partially explain the elevated HDL levels in USF1 deficient mice. Expression of NF-κB, a key regulator of inflammation was decreased in peritoneal macrophages from USF1 deficient mice and, furthermore, secretion of cytokines (MCP-1 and IL-1β) was found to be decreased in USF1 silenced THP-1 macrophages. Arrows denote the effects of USF1 deficiency at relevant sites

In this study we have identified USF1 as a novel factor affecting HDL functionality, thus having an impact on HDL metabolism beyond its effects on the levels of HDL-C. The majority of epidemiological studies of HDL functionality have recently focused on macrophage cholesterol efflux [7, 8]. These studies have shown that cholesterol efflux is inversely proportional to CVD risk, and demonstrated cholesterol efflux to be a better CVD risk predictor than HDL-C levels. Thus, cholesterol efflux facilitated by HDL merits further study, and factors boosting cholesterol efflux could potentially provide antiatherogenic effects. Based on our study, USF1 appears to be such factor, regulating the cholesterol acceptor properties of HDL particles, such their phospholipid (PL) content. Moreover, we show evidence that USF1 modulates the expression of cholesterol transporters (SR-A1, ABCA1), and gene expression of critical metabolic pathways of intracellular cholesterol ester hydrolyzing and esterifying enzymes crucial for the intracellular cholesterol turnover in macrophages. These observations are summarized in Fig. 5 and discussed below.

First, *USF1* silencing activated cholesterol removal from macrophages. This enhancement in cholesterol release capacity was most likely due to enhanced ABCA1-mediated cholesterol efflux. The results agree well with previous studies in which USF1 was identified as a repressor of *ABCA1* [19, 32]. The expression of *SCARB1*, a passive transporter, was unchanged, while the mRNA level of *ABCG1* was decreased. It is possible that ABCG1 expression could be reduced in response to the change in cholesterol status of the cells, as ABCA1 expression has been increased by a mechanism that may not affect ABCG1. This finding requires further study. The observations suggest that upon USF1 deficiency, ABCA1 mediated cholesterol efflux is the major pathway responsible for net macrophage cholesterol efflux. As ABCA1 protein was increased in USF1 silenced hepatocytes, USF1 deficiency could also contribute to elevated plasma HDL-C levels by enhancing secretion of nascent HDL particles from the liver.

Second, USF1 deficiency improved macrophage cholesterol efflux also through conferring enhanced cholesterol acceptor capacity to serum and HDL particles. The *Usf1*^*−/−*^-derived HDL particles exhibited higher phospholipid content than those of *Usf1*^*+/+*^ mice, and since Fournier et al. have previously shown that HDL phospholipid content is the major factor determining HDL-dependent cholesterol efflux [22], our results point to elevated phospholipid content being one important factor responsible for the enhanced acceptor capacity of *Usf1*^*−/−*^-derived HDL particles (Fig. 5).

Third, USF1 deficiency increased the gene expression of intracellular enzymes affecting macrophage cholesterol metabolism. Of these enzymes, LIPA and NCEH1 are responsible for cholesterol ester hydrolysis, releasing unesterified cholesterol and fatty acids, and ACAT1 for re-esterification of cholesterol. High levels of NCEH1 and LIPA have been shown to protect against atherosclerosis [28, 31], and they are associated with increased cholesterol efflux [27, 30]. The upregulation of these mRNAs potentially indicates a higher rate of cholesterol trafficking in the cell, resulting in enhanced cellular cholesterol transfer to acceptors (Fig. 5). However, in radiolabeling experiments, we did not observe a significant difference in cholesterol esterification or [^14^C]-cholesterol distribution between unesterified and esterified pools between USF1 deficient and control cells. This is consistent with a model in which the intracellular turnover of cholesterol in USF1 deficient cells is enhanced, making it more readily available for ABCA1-mediated efflux to HDL acceptors (Fig. 5).

*Usf1* deficiency protected against LPS-induced increase in cholesterol deposition into macrophages. LPS has been implicated in human cardiovascular disease [33], and is thought to contribute to the development of arterial plaques through activation of pro-inflammatory pathways [34]. The chronic low-grade inflammation associated with dyslipidemia predisposes to atherosclerosis via the formation of foam cells. Thus, the atheroprotective effect of *Usf1* deficiency in *Ldlr*^*−/−*^ background [16] could be partly mediated by the attenuated formation of foam cells despite the severe hyperlipidemia observed in *Ldlr*^*−/−*^ background. The reduced cholesterol accumulation in the present study is likely explained by the reduction in scavenger receptor SR-A1 (gene *MSR1*) whose mRNA expression was decreased following USF1 depletion. On the other hand, the mRNA expression of *CD36*, another scavenger receptor, was elevated. While this finding may first seem contradictory, it appears more plausible considering that CD36 is a marker of anti-inflammatory M2 macrophages [35]. In fact, as the expression of NF-κB was down-regulated in USF1 deficient macrophages as well as the secretion of pro-inflammatory cytokines MCP-1 and IL-1β, increased *CD36* expression is well in line with the general anti-inflammatory status conferred by *Usf1* deficiency [16]. The anti-inflammatory environment most likely repels cholesterol accumulation into macrophages.

This study, showing that *Usf1* deficiency enhances cholesterol efflux, lowers macrophage inflammatory status, and reduces cholesterol accumulation, supports and expands our previous findings, in which we showed that *Usf1* deficiency results in higher HDL-C, lower triglycerides, and protection against diet-induced obesity, insulin resistance, systemic inflammation, and atherosclerosis [16]. Mice lacking *Usf1* on *Ldlr*^*−/−*^ background displayed a 4-fold reduction in atherosclerosis, and human individuals harboring an allele associated with 18% reduction in *USF1* mRNA displayed a 45% reduction in plaque area in coronary arteries as well as 47% reduction in plaque calcification. While the causal relationship between elevated cholesterol efflux and CVD events remains to be proven in future studies, our study is well in line with epidemiological reports showing enhanced cholesterol efflux capacity to be associated with reduction in CVD events [7], and provides further support for the importance of functional improvement of HDL particles regarding atheroprotection. The present study includes some important limitations: It is possible that USF1 regulates macrophage polarization between M1 and M2, which could affect the results. Further, although we report an elevated total phospholipid content in HDL derived from *Usf1* deficient mice, the mechanism behind this observation remains unknown. Finally, thorough HDL phospholipidomics and proteomics analyses would be relevant in order to address in more detail the impact of *Usf1* deficiency on HDL function in cholesterol efflux. These issues are important goals for the future studies.

## Conclusions

We have identified USF1 as a novel factor affecting macrophage inflammatory status and cholesterol homeostasis, whose deficiency not only raises HDL-C levels, but, more importantly, improves HDL functionality and reduces inflammation.

## Abbreviations

ABCA1: ATP-binding Cassette Transporter A1
ACAT1: Acyl-CoA: Cholesterol Acyltransferase 1
apoA-I: apolipoprotein A-I
C: Cholesterol
CE: Cholesterol ester
CVD: Cardiovascular disease
HDL-C: High-density lipoprotein cholesterol
IL-1β: Interleukin 1β
LAL: Lysosomal acid lipase
LDL: Low-density lipoprotein
LPS: Lipopolysaccharide
MCP-1: Monocyte chemoattractant protein 1
NCEH1: Neutral cholesterol ester hydrolase 1
NF-κB: Nuclear factor- κB
PL: Phospholipids
SR-A1: Scavenger receptor A1
USF1: Upstream stimulatory factor 1

## References

[1] Robinson JG, Stone NJ (2015). The 2013 ACC/AHA guideline on the treatment of blood cholesterol to reduce atherosclerotic cardiovascular disease risk: a new paradigm supported by more evidence. Eur Heart J.

[2] Barter PJ, Caulfield M, Eriksson M, Grundy SM, Kastelein JJ, Komajda M, Lopez-Sendon J, Mosca L, Tardif JC, Waters DD, Shear CL, Revkin JH, Buhr KA, Fisher MR, Tall AR, Brewer B, Investigators ILLUMINATE (2007). Effects of torcetrapib in patients at high risk for coronary events. N Engl J Med.

[3] Landray MJ, Haynes R, Hopewell JC, Parish S, Aung T, Tomson J, Wallendszus K, Craig M, Jiang L, Collins R, Armitage J, HPS2-THRIVE Collaborative Group (2014). Effects of extended-release niacin with laropiprant in high-risk patients. N Engl J Med.

[4] Investigators AIM-HIGH, Boden WE, Probstfield JL, Anderson T, Chaitman BR, Desvignes-Nickens P, Koprowicz K, McBride R, Teo K, Weintraub W (2011). Niacin in patients with low HDL cholesterol levels receiving intensive statin therapy. N Engl J Med.

[5] Voight BF, Peloso GM, Orho-Melander M, Frikke-Schmidt R, Barbalic M, Jensen MK, Hindy G, Holm H, Ding EL, Johnson T, Schunkert H, Samani NJ, Clarke R, Hopewell JC, Thompson JF, Li M, Thorleifsson G, Newton-Cheh C, Musunuru K, Pirruccello JP, Saleheen D, Chen L, Stewart A, Schillert A, Thorsteinsdottir U, Thorgeirsson G, Anand S, Engert JC, Morgan T, Spertus J, Stoll M, Berger K, Martinelli N, Girelli D, McKeown PP, Patterson CC, Epstein SE, Devaney J, Burnett MS, Mooser V, Ripatti S, Surakka I, Nieminen MS, Sinisalo J, Lokki ML, Perola M, Havulinna A, de Faire U, Gigante B, Ingelsson E, Zeller T, Wild P, de Bakker PI, Klungel OH, Maitland-van der Zee AH, Peters BJ, de Boer A, Grobbee DE, Kamphuisen PW, Deneer VH, Elbers CC, Onland-Moret NC, Hofker MH, Wijmenga C, Verschuren WM, Boer JM, van der Schouw YT, Rasheed A, Frossard P, Demissie S, Willer C, Do R, Ordovas JM, Abecasis GR, Boehnke M, Mohlke KL, Daly MJ, Guiducci C, Burtt NP, Surti A, Gonzalez E, Purcell S, Gabriel S, Marrugat J, Peden J, Erdmann J, Diemert P, Willenborg C, Konig IR, Fischer M, Hengstenberg C, Ziegler A, Buysschaert I, Lambrechts D, Van de Werf F, Fox KA, El Mokhtari NE, Rubin D, Schrezenmeir J, Schreiber S, Schafer A, Danesh J, Blankenberg S, Roberts R, McPherson R, Watkins H, Hall AS, Overvad K, Rimm E, Boerwinkle E, Tybjaerg-Hansen A, Cupples LA, Reilly MP, Melander O, Mannucci PM, Ardissino D, Siscovick D, Elosua R, Stefansson K, O'Donnell CJ, Salomaa V, Rader DJ, Peltonen L, Schwartz SM, Altshuler D, Kathiresan S (2012). Plasma HDL cholesterol and risk of myocardial infarction: a mendelian randomisation study. Lancet.

[6] Toth PP, Barter PJ, Rosenson RS, Boden WE, Chapman MJ, Cuchel M, D'Agostino S, RB MHD, Davidson WS, Heinecke JW, Karas RH, Kontush A, Krauss RM, Miller M, Rader DJ (2013). High-density lipoproteins: a consensus statement from the National Lipid Association. J Clin Lipidol.

[7] Khera AV, Cuchel M, de la Llera-Moya M, Rodrigues A, Burke MF, Jafri K, French BC, Phillips JA, Mucksavage ML, Wilensky RL, Mohler ER, Rothblat GH, Rader DJ (2011). Cholesterol efflux capacity, high-density lipoprotein function, and atherosclerosis. N Engl J Med.

[8] Rohatgi A, Khera A, Berry JD, Givens EG, Ayers CR, Wedin KE, Neeland IJ, Yuhanna IS, Rader DR, de Lemos JA, Shaul PW (2014). HDL cholesterol efflux capacity and incident cardiovascular events. N Engl J Med.

[9] Eckardstein A (2012). Tachometer for reverse cholesterol transport?. J Am Heart Assoc.

[10] Pajukanta P, Lilja HE, Sinsheimer JS, Cantor RM, Lusis AJ, Gentile M, Duan XJ, Soro-Paavonen A, Naukkarinen J, Saarela J, Laakso M, Ehnholm C, Taskinen MR, Peltonen L (2004). Familial combined hyperlipidemia is associated with upstream transcription factor 1 (USF1). Nat Genet.

[11] Laurila PP, Naukkarinen J, Kristiansson K, Ripatti S, Kauttu T, Silander K, Salomaa V, Perola M, Karhunen PJ, Barter PJ, Ehnholm C, Peltonen L (2010). Genetic association and interaction analysis of USF1 and APOA5 on lipid levels and atherosclerosis. Arterioscler Thromb Vasc Biol.

[12] Putt W, Palmen J, Nicaud V, Tregouet DA, Tahri-Daizadeh N, Flavell DM, Humphries SE, Talmud PJ (2004). Variation in USF1 shows haplotype effects, gene : gene and gene : environment associations with glucose and lipid parameters in the European atherosclerosis research study II. Hum Mol Genet.

[13] Huertas-Vazquez A, Aguilar-Salinas C, Lusis AJ, Cantor RM, Canizales-Quinteros S, Lee JC, Mariana-Nunez L, Riba-Ramirez RM, Jokiaho A, Tusie-Luna T, Pajukanta P (2005). Familial combined hyperlipidemia in Mexicans: association with upstream transcription factor 1 and linkage on chromosome 16q24.1. Arterioscler Thromb Vasc Biol.

[14] Kristiansson K, Ilveskoski E, Lehtimaki T, Peltonen L, Perola M, Karhunen PJ (2008). Association analysis of allelic variants of USF1 in coronary atherosclerosis. Arterioscler Thromb Vasc Biol.

[15] Komulainen K, Alanne M, Auro K, Kilpikari R, Pajukanta P, Saarela J, Ellonen P, Salminen K, Kulathinal S, Kuulasmaa K, Silander K, Salomaa V, Perola M, Peltonen L (2006). Risk alleles of USF1 gene predict cardiovascular disease of women in two prospective studies. PLoS Genet.

[16] Laurila PP, Soronen J, Kooijman S, Forsstrom S, Boon MR, Surakka I, Kaiharju E, Coomans CP, Van Den Berg SA, Autio A, Sarin AP, Kettunen J, Tikkanen E, Manninen T, Metso J, Silvennoinen R, Merikanto K, Ruuth M, Perttila J, Makela A, Isomi A, Tuomainen AM, Tikka A, Ramadan UA, Seppala I, Lehtimaki T, Eriksson J, Havulinna A, Jula A, Karhunen PJ, Salomaa V, Perola M, Ehnholm C, Lee-Rueckert M, Van Eck M, Roivainen A, Taskinen MR, Peltonen L, Mervaala E, Jalanko A, Hohtola E, Olkkonen VM, Ripatti S, Kovanen PT, Rensen PC, Suomalainen A, Jauhiainen M (2016). USF1 deficiency activates brown adipose tissue and improves cardiometabolic health. Sci Transl Med.

[17] van Haperen R, van Tol A, Vermeulen P, Jauhiainen M, van Gent T, van den Berg P, Ehnholm S, Grosveld F, vand der Kamp A, de Crom R (2000). Human plasma phospholipid transfer protein increases the antiatherogenic potential of high density lipoproteins in transgenic mice. Arterioscler Thromb Vasc Biol.

[18] Wu Z, Irizarry R, Gentleman R, Martinez-Murillo F, Spencer F (2004). A model-based background adjustment for oligonucleotide expression arrays. J Am Stat Assoc.

[19] Yang XP, Freeman LA, Knapper CL, Amar MJ, Remaley A, Brewer HB, Santamarina-Fojo S (2002). The E-box motif in the proximal ABCA1 promoter mediates transcriptional repression of the ABCA1 gene. J Lipid Res.

[20] Yetukuri L, Soderlund S, Koivuniemi A, Seppanen-Laakso T, Niemela PS, Hyvonen M, Taskinen MR, Vattulainen I, Jauhiainen M, Oresic M (2010). Composition and lipid spatial distribution of HDL particles in subjects with low and high HDL-cholesterol. J Lipid Res.

[21] Laurila PP, Surakka I, Sarin AP, Yetukuri L, Hyotylainen T, Soderlund S, Naukkarinen J, Tang J, Kettunen J, Mirel DB, Soronen J, Lehtimaki T, Ruokonen A, Ehnholm C, Eriksson JG, Salomaa V, Jula A, Raitakari OT, Jarvelin MR, Palotie A, Peltonen L, Oresic M, Jauhiainen M, Taskinen MR, Ripatti S (2013). Genomic, transcriptomic, and lipidomic profiling highlights the role of inflammation in individuals with low high-density lipoprotein cholesterol. Arterioscler Thromb Vasc Biol.

[22] Fournier, N., M. de la Llera Moya, B. F. Burkey, J. B. Swaney, J. Paterniti Jr, N. Moatti, V. Atger, and G. H. Rothblat. 1996. Role of HDL phospholipid in efflux of cell cholesterol to whole serum: studies with human apoA-I transgenic rats. J Lipid Res 37**:** 1704–1711. 8864954

[23] Gelissen IC, Harris M, Rye KA, Quinn C, Brown AJ, Kockx M, Cartland S, Packianathan M, Kritharides L, Jessup W (2006). ABCA1 and ABCG1 synergize to mediate cholesterol export to apoA-I. Arterioscler Thromb Vasc Biol.

[24] Yu XH, Fu YC, Zhang DW, Yin K, Tang CK (2013). Foam cells in atherosclerosis. Clin Chim Acta.

[25] Kunjathoor VV, Febbraio M, Podrez EA, Moore KJ, Andersson L, Koehn S, Rhee JS, Silverstein R, Hoff HF, Freeman MW (2002). Scavenger receptors class A-I/II and CD36 are the principal receptors responsible for the uptake of modified low density lipoprotein leading to lipid loading in macrophages. J Biol Chem.

[26] Ouimet M, Franklin V, Mak E, Liao X, Tabas I, Marcel YL (2011). Autophagy regulates cholesterol efflux from macrophage foam cells via lysosomal acid lipase. Cell Metab.

[27] Du H, Grabowski GA (2004). Lysosomal acid lipase and atherosclerosis. Curr Opin Lipidol.

[28] Du H, Schiavi S, Wan N, Levine M, Witte DP, Grabowski GA (2004). Reduction of atherosclerotic plaques by lysosomal acid lipase supplementation. Arterioscler Thromb Vasc Biol.

[29] Dove DE, Su YR, Zhang W, Jerome WG, Swift LL, Linton MF, Fazio S (2005). ACAT1 deficiency disrupts cholesterol efflux and alters cellular morphology in macrophages. Arterioscler Thromb Vasc Biol.

[30] Igarashi M, Osuga J, Uozaki H, Sekiya M, Nagashima S, Takahashi M, Takase S, Takanashi M, Li Y, Ohta K, Kumagai M, Nishi M, Hosokawa M, Fledelius C, Jacobsen P, Yagyu H, Fukayama M, Nagai R, Kadowaki T, Ohashi K, Ishibashi S (2010). The critical role of neutral cholesterol ester hydrolase 1 in cholesterol removal from human macrophages. Circ Res.

[31] Sekiya M, Osuga J, Nagashima S, Ohshiro T, Igarashi M, Okazaki H, Takahashi M, Tazoe F, Wada T, Ohta K, Takanashi M, Kumagai M, Nishi M, Takase S, Yahagi N, Yagyu H, Ohashi K, Nagai R, Kadowaki T, Furukawa Y, Ishibashi S (2009). Ablation of neutral cholesterol ester hydrolase 1 accelerates atherosclerosis. Cell Metab.

[32] Pan X, Jiang XC, Hussain MM (2013). Impaired cholesterol metabolism and enhanced atherosclerosis in clock mutant mice. Circulation.

[33] Kallio KA, Buhlin K, Jauhiainen M, Keva R, Tuomainen AM, Klinge B, Gustafsson A, Pussinen PJ (2008). Lipopolysaccharide associates with pro-atherogenic lipoproteins in periodontitis patients. Innate Immun.

[34] Lehr HA, Sagban TA, Ihling C, Zahringer U, Hungerer KD, Blumrich M, Reifenberg K, Bhakdi S (2001). Immunopathogenesis of atherosclerosis: endotoxin accelerates atherosclerosis in rabbits on hypercholesterolemic diet. Circulation.

[35] Huang SC, Everts B, Ivanova Y, O'Sullivan D, Nascimento M, Smith AM, Beatty W, Love-Gregory L, Lam WY, O'Neill CM, Yan C, Du H, Abumrad NA, Urban JF, Artyomov MN, Pearce EL, Pearce EJ (2014). Cell-intrinsic lysosomal lipolysis is essential for alternative activation of macrophages. Nat Immunol.

